# Preheating colistimethate sodium enhances its efficacy against multi/extensively drug-resistant Gram-negative bacilli in preservation fluid

**DOI:** 10.1038/s41598-026-38748-5

**Published:** 2026-02-05

**Authors:** Daqian Tang, Lijie Zhang, Yazhe Duan, Yanhua Li, Yuhong Li, Pei Zhang, Yuxiang Wan, Kang Wu, Wenyu Zhao, Junhao Yu, Li Zeng, Mingxing Sui

**Affiliations:** 1 Department of Organ Transplantation, Shanghai Changhai Hospital, The First Affiliated Hospital of Naval Medical University, Shanghai, China; 2 Clinical Laboratory, Shanghai Changhai Hospital, The First Affiliated Hospital of Naval Medical University, Shanghai, China; 3 Department of Urology, No 908th hospital of Chinese PLA Joint Logistic Support Force, Nanchang, Jiangxi China

**Keywords:** Colistimethate sodium, Preservation fluid, Multidrug resistant, Extensively drug-resistant, Gram-negative bacteria, Organ transplantation, Decontamination, Static cold storage, Medical research, Microbiology

## Abstract

This study aimed to evaluate the efficacy and preliminary safety of preheated colistimethate sodium (CMS) in reducing the load of multidrug-resistant and extensively drug-resistant (MDR/XDR) Gram-negative bacilli in preservation fluid (PF) under simulated static cold storage (SCS) conditions in vitro. CMS was preheated at 37–60 °C for 5 ~ 30 min. The antibacterial effects of preheated CMS were evaluated against five MDR/XDR strains: carbapenem-resistant *Pseudomonas aeruginosa* (CRPA), *Klebsiella pneumoniae* (CRKP), *Escherichia coli* (CREC), *Acinetobacter baumannii* (CRAB), and methicillin-resistant *Staphylococcus aureus* (MRSA). Bacterial counts were assessed after 3 h of SCS, and the average inhibition rate (AIR) was calculated. The safety experiment was performed to evaluate the nephrotoxicity of preheated CMS under hypothermic condition. Preheating CMS at 60 °C for 15 ~ 30 min significantly reduced bacterial loads of CRPA, CRKP, CREC, and CRAB, with average inhibition rates (AIR) up to 100%, 96.91%, 98.36%, and 85.06%, respectively. At 37 °C, extended heating (30 min) was required for partial efficacy against CRKP and CRAB, while CREC remained largely unaffected. MRSA showed no susceptibility to preheated CMS. Preheated CMS did not cause significant morphological alterations or reduction in HK-2 cell viability within 3 ~ 6 h of SCS. Thermal pretreatment of CMS at 60 °C represents a novel, practical, and safe strategy for PF decontamination, providing rapid bactericidal activity against frequently encountered MDR/XDR Gram-negative bacilli during the SCS process.

## Introduction

Organ transplantation is frequently complicated by early postoperative infections^1^, and contaminated preservation fluid (PF) has been strongly correlated with these early infections^2–4^. Positive PF cultures are commonly reported, with positive rates ranging from approximately 9.5% to 20.5% in retrospective studies and reaching 62.5% to 98% in prospective studies^5^. Although PF contamination is relatively common, the overall incidence of preservation fluid-related infections (PFRI) remains low (approximately 1.5%), likely attributable to low microbial burden and the efficacy of prophylactic antibiotics against most organisms^6,7^. However, the high-risk pathogens, particularly Gram-negative bacteria (GNB), account for 15.8% to 84.3% among culture-positive samples, substantially increasing the risk of PFRI^2,8^. Conventional antibiotic prophylaxis is frequently ineffective against multidrug-resistant (MDR) or extensively drug-resistant (XDR) organisms [7–9]. The transmission of MDR/XDR GNB pathogens is associated with significant morbidity in transplant recipients^9^, contributing to prolonged hospitalization, intensive care admission, delayed graft function, graft loss, and elevated mortality^10,11^. Given the increasing prevalence of MDR/XDR pathogens and the potential inadequacy of conventional prophylaxis^12–14^, a major clinical challenge is to proactively reduce the microbial load in preservation fluid and prevent transmission of high-risk organisms.

Colistimethate sodium (CMS) is one of the first-line agents against MDR-GNB infections with extensive clinical evidence, holding great potential value in the graft decontamination in organ transplantation. As an inactive prodrug, CMS undergoes hydrolysis to form its active metabolite, colistin, with antibacterial activity quantified in colistin base activity (CBA) units and demonstrated efficacy against MDR-GNB^15–17^. In vitro, the hydrolysis of CMS is predominantly temperature-dependent, achieving complete conversion to colistin within 12 ~ 24 h in aqueous solution at 37 °C ^18^. Based on these characteristics, we propose that adding preheated CMS to PF during static cold storage (SCS) could reduce the burden of MDR/XDR bacteria. This approach aims to provide experimental evidence and safety data for this decontamination protocol to decrease the incidence of PFRI.

## Results

### Main effect of CMS preheating temperature

**Fig. 1:**
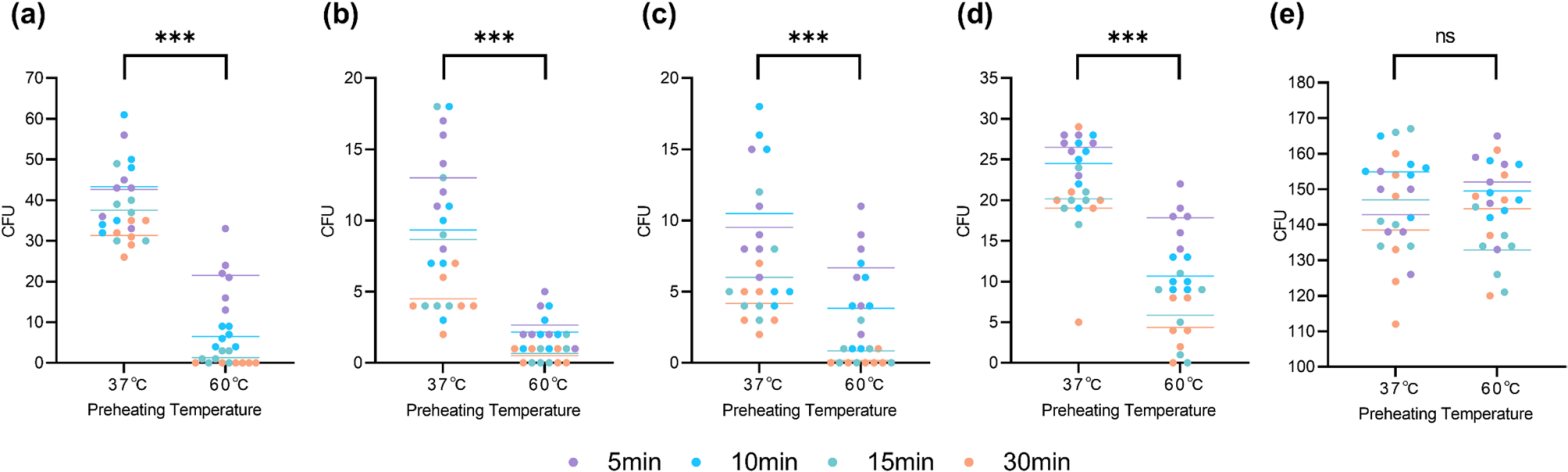
Effect of CMS preheating temperature on bacterial colony counts. **(a)** CRPA. **(b)** CRKP. **(c)** CREC. **(d)** CRAB. **(e)** MRSA. ***P < 0.001; ns, not significant.

As the preheating temperature of CMS increased from 37 °C to 60 °C, a significant main effect of temperature was observed, leading to a substantial reduction in the colony counts of CRPA, CRKP, CREC, and CRAB (all *P* values < 0.001; Fig. 1a–d). In contrast, no significant effect was observed on MRSA colony counts (*P* = 0.761; Fig. 1e).

### Main effect of CMS preheating duration

**Fig. 2:**
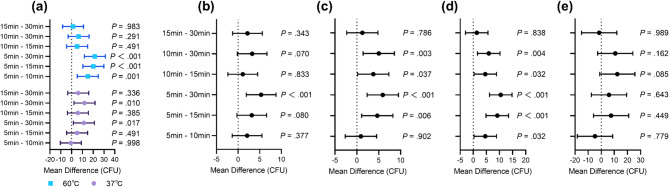
Effect of CMS preheating duration on bacterial colony counts with 95% confidence intervals. **(a)** CRPA. **(b)** CRKP. **(c)** CREC. **(d)** CRAB. **(e)** MRSA.

For CRPA, a significant interaction was identified between preheating temperature and time (*P* = 0.014; data not shown), indicating a synergistic effect of these two factors on reducing the bacterial load. Simple effects analysis for heating duration showed that at 60 °C, significant differences were observed between the 5-minute group and each of the longer duration groups (15–30 min). However, at 37 °C, a minimum of 30 min of heating was required to achieve a statistically significant difference (Fig. 2a).

For CRKP, CREC, and CRAB, no significant interaction between preheating temperature and time was observed (*P* = 0.118, 0.527, and 0.214, respectively; data not shown), suggesting that the effects of temperature and time on reducing colony counts were independent. Post hoc analysis for heating duration revealed that 30 min of preheating resulted in a significant reduction compared to 5 min (Fig. 2b–d). Additionally, for CREC and CRAB, a significant difference was also observed between 15 min and 5 min of preheating (Fig. 2c–d). In contrast, preheating duration did not result in significant changes in colony counts for MRSA across any of the tested time points (Fig. 2e).

### Efficacy of preheated CMS against each bacterial strain

**Fig. 3:**
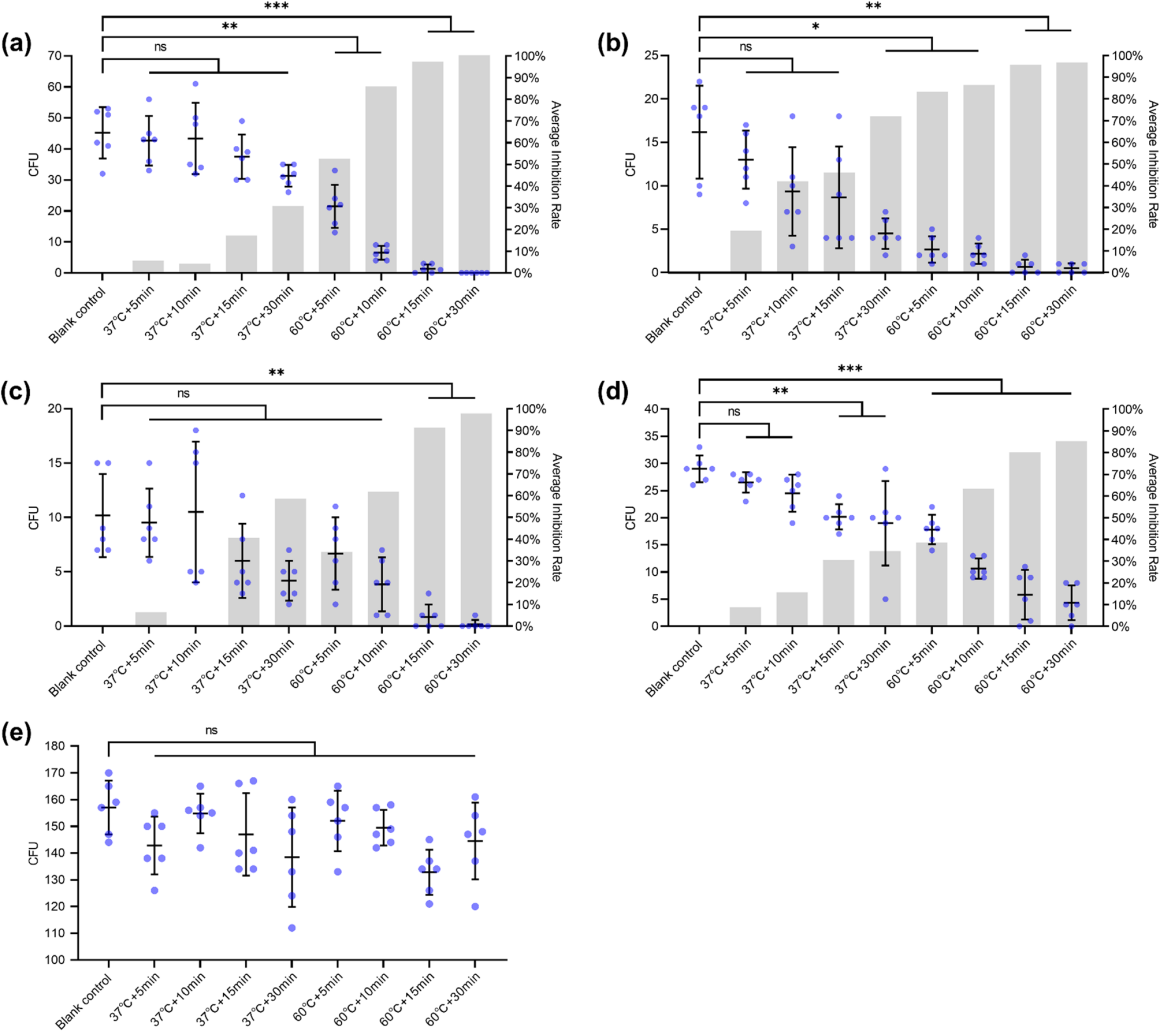
Antibacterial activity of preheated CMS. Colony-forming units and average inhibition rates are shown for each strain compared to blank control. **(a)** CRPA (Welch’s ANOVA with Games–Howell test). **(b)** CRKP (Welch’s ANOVA with Games–Howell test). **(c)** CREC (Kruskal–Wallis H test with Dunn’s test). **(d)** CRAB (one-way ANOVA with Tukey’s test). **(e)** MRSA (Kruskal–Wallis H test). *P < 0.05, **P < 0.01, ***P < 0.001; ns, not significant.

Preheating CMS at 60 °C significantly reduced bacterial loads across all Gram-negative strains compared to the blank control (Fig. 3a-d). Near-complete eradication of CRPA was observed after 15–30 min at 60 °C, with AIR values of 97.04% and 100%, respectively. In contrast, preheating at 37 °C for up to 30 min did not significantly reduce CRPA (max AIR: 30.63%; Fig. 3a).

For CRKP, significant reduction required at least 30 min at 37 °C (AIR: 72.17%), though all 60 °C groups showed higher efficacy (AIR: 83.51–96.91%; Fig. 3b). CREC was significantly reduced only after 15–30 min at 60 °C (AIR: 91.81% and 98.36%, respectively; Fig. 3c). CRAB showed significant reduction after 15–30 min at 37 ℃, but these effects were surpassed even by the 5-minute 60 °C group. The highest AIR for CRAB was 85.06% in the 60 °C groups (Fig. 3d). No significant reduction was observed for MRSA under any condition (Fig. 3e).

### Cytotoxicity of preheated CMS on HK-2 cells

**Fig. 4:**
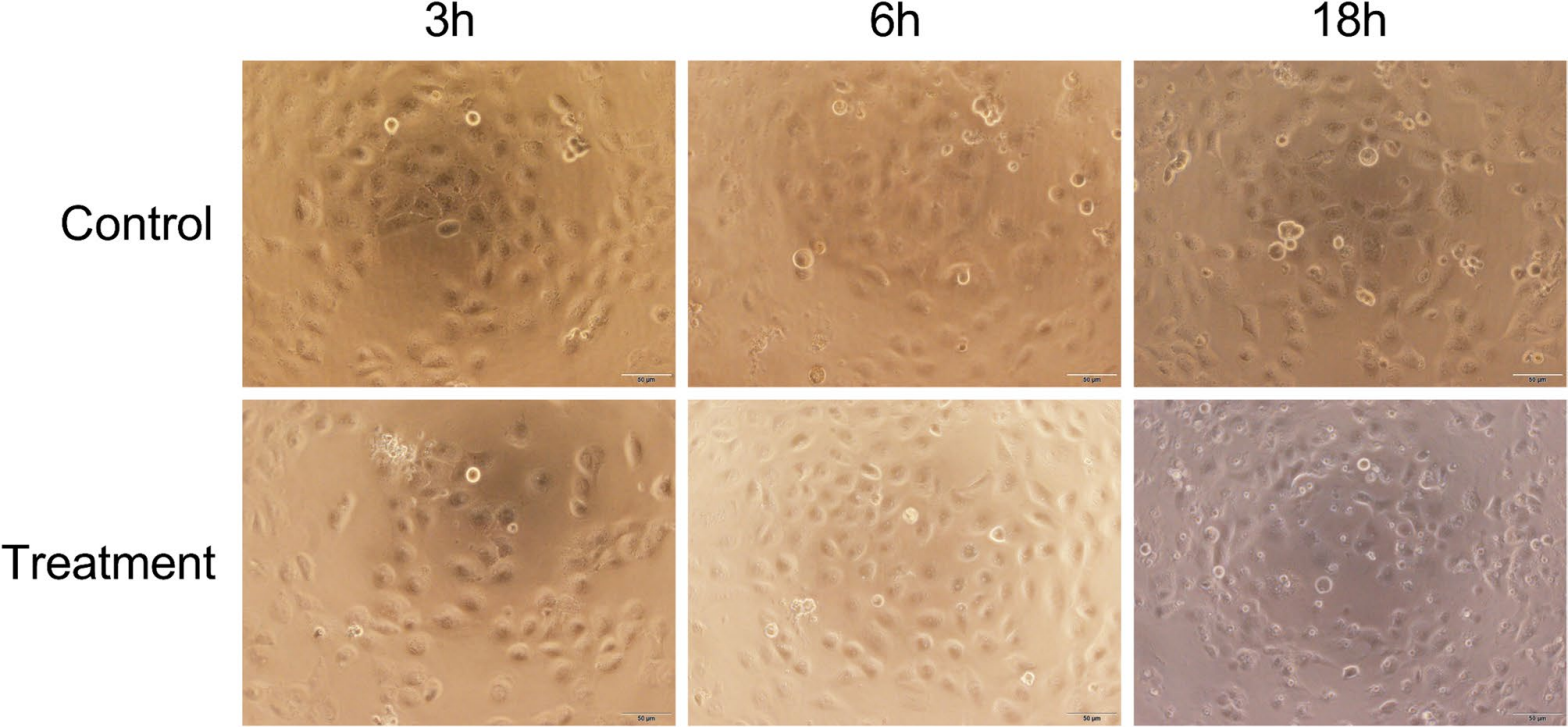
Morphology of HK-2 cells after exposure to preheated CMS during hypothermic storage. Representative inverted light micrographs (40×) of HK-2 cells following 3, 6, or 18 h incubation at 0 ~ 4 °C in serum-free medium (Control) or serum-free medium supplemented with preheated CMS (Treatment). Scale bar, 50 μm.

**Fig. 5:**
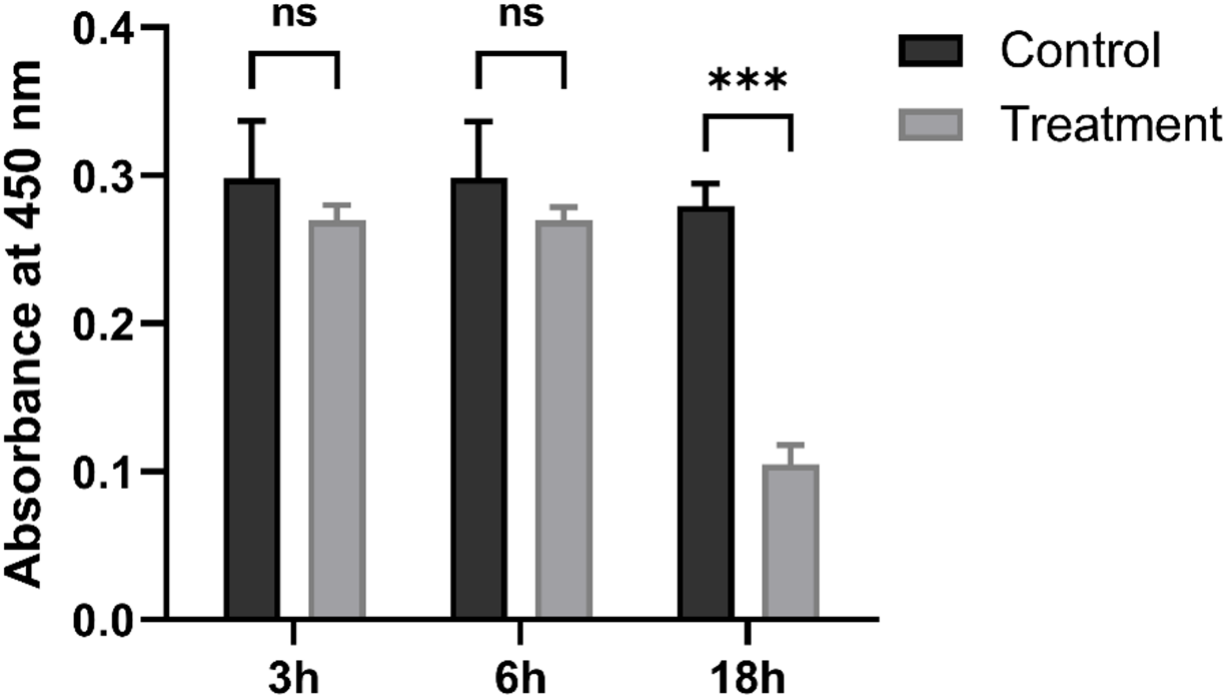
Viability of HK-2 cells after exposure to preheated CMS during hypothermic incubation. Cell viability was assessed by the CCK-8 assay and presented as background-subtracted absorbance at 450 nm after 3, 6, or 18 h at 0 ~ 4 °C. ***P < 0.001; ns, not significant (independent-samples t-test).

After 3–6 h of exposure to preheated CMS under SCS conditions, HK-2 cells remained similar cell shape, confluence, and adherence to the control group (Fig. 4). The CCK-8 assay showed comparable corrected OD_450_ values between the preheated CMS group and the control at 3 h (0.271 ± 0.011 vs. 0.288 ± 0.039, *P* = 0.286) and at 6 h (0.277 ± 0.009 vs. 0.294 ± 0.036, *P* = 0.269) (Fig. 5). After 18 h, however, CMS-treated cells presented clear evidence of damage, including lower cell density, increased cellular debris, and a higher proportion of irregular morphology (Fig. 4). The corrected OD_450_​ was also markedly reduced in the CMS group (0.096 ± 0.012 vs. 0.284 ± 0.015, *P* < 0.001) (Fig. 5).

## Discussion

Adding antibiotics to PF during organ perfusion and storage is an increasingly recognized strategy, as it enables graft decontamination through in vitro intervention. The increasing prevalence of MDR-GNB has renewed clinical interest in polymyxins as last-line therapeutic agents^19,20^. Our previous studies have shown that the decontamination regimen based on colistin sulfate reduces early postoperative infections caused by MDR-GNB^21^. However, the clinical availability of colistin sulfate is much lower than that of CMS, which is more widely marketed globally. Studies have shown that the in vitro antibacterial activity of CMS is influenced by factors such as solution temperature, concentration, and solvent composition^22,23^, which may limit its utility in PF decontamination. To overcome these limitations, we investigated the hydrolytic conversion and in vitro antibacterial effects of CMS in PF at 0–4℃ while varying the preheating temperature and duration.

Our findings indicate that thermal pretreatment substantially enhances the antibacterial activity of CMS. Preheating in a 60 °C water bath for 5 min resulted in significant growth inhibition against CRKP, CRAB, and CRPA. For CREC, a minimum of 15 min at 60 °C was necessary to achieve significant antibacterial efficacy. Extending the heating duration to 30 min further enhanced bacterial inhibition, resulting in near-complete eradication of CRKP, CREC, and CRPA. Consistent with known mechanisms, our findings support the intrinsic resistance of Gram-positive bacteria to colistin, as they lack the outer membrane lipopolysaccharide targeted by this antibiotic. Based on these results, we propose that the CMS be preheated at 60 °C for 15 ~ 30 min before being added to the PF to reduce the burden of MDR/XDR-GNB, thereby potentially leading to fewer early postoperative infections and improved transplant-related outcomes.

The hydrolysis of CMS is influenced by multiple factors, including solvent, solute, temperature, and pH. Previous studies have demonstrate that both CMS and colistin remain highly stable in water (pH 5.63 ~ 5.98) at 4 °C ^18^. The extent of CMS hydrolysis to colistin was found to be less than 0.1% after more than seven days of hypothermic storage^23^, while over 97.4% of colistin remained stable after 60 days of storage^18^. In this study, CMS was preheated for activation and then cooled to 0–4 °C to stabilize the activated colistin levels. However, solvent used in our experiment was hypertonic adenine citrate solution (mainly containing potassium citrate, sodium citrate, magnesium sulfate, mannitol, and adenine phosphate, pH 7.0 ± 0.10). Although studies on CMS hydrolysis and stability in such solvents are few reported, our results confirm that the extent of CMS hydrolysis to colistin was primarily determined by preheating temperature and duration.

Polymyxins are clinically associated with nephrotoxicity possibly related to (i) uptake of the drug by proximal tubular epithelial cells via transporters such as Megalin^24^, OCTN2 ^25^, and PEPT2 ^26^, and (ii) excessive time and dose exposure due to prolonged treatment duration and cumulative dose^27^. Intracellular accumulation can promote oxidative stress, inflammation, and apoptosis, leading to tubular cell injury and impaired renal function^28^. In this hypothermic model, active transport is strongly suppressed; consequently, 3–6 h exposure to preheated CMS did not measurably compromise HK-2 cell morphology or viability, with corrected OD_450_​ values similar to the control (both *P* > 0.05), whereas 18 h exposure led to clear evidence of injury.

In conclusion, thermal pretreatment of CMS at 60 °C represents a novel, practical, and safe strategy for PF decontamination, providing rapid bactericidal activity against frequently encountered MDR/XDR Gram-negative bacilli during the < 6 h SCS process.

This study primarily focused on the decontamination against four major MDR/XDR-GNB, leaving ample scope for further research. Firstly, active perfusion improves antibiotic delivery to the graft and, via continuous flow and shear stress, helps remove adherent intravascular bacteria^29^. Therefore, a combined approach integrating preheated CMS (60 °C for 15 ~ 30 min) with perfusion could be adopted for enhanced graft decontamination. Secondly, antibiotics against Gram-positive bacteria and fungi might be incorporated if warranted. These integrated strategies hold the potential to reduce the incidence of PFRI caused by MDR/XDR pathogens and increase the utilization rate of available donor organs. Moreover, the 3-hour decontamination protocol aligns well with clinical organ preservation and transportation timelines, minimizing the risk of significant delay in the transplantation process and avoiding the adverse effect on graft outcomes due to prolonged cold ischemia time. Prophylactic antibiotic overuse may contribute to the development of antimicrobial resistance, which is particularly concerning given the global emergence of colistin-resistant strains^30,31^. However, in this study CMS was administered as a single high-dose exposure in vitro, which is expected to reduce the risk of resistance selection.

This study has several limitations. Firstly, the sample size in each experimental group was limited to six replicates. Secondly, the experimental conditions were confined to two temperatures (37 °C and 60 °C) and four heating durations (ranging from 5 to 30 min). Future studies should incorporate broader temperature and time gradients, along with larger sample sizes, to obtain more reliable and precise treatment parameters. Additionally, the study did not quantify the generation of CBA from heated CMS, leaving the relationship between conversion efficiency unclear, nor did it evaluate differences between various CMS products.

## Methods

### Materials

CMS for injection (150 mg, expressed as colistin E) was obtained from CHIA TAI Tianqing Pharmaceutical Group Co., Ltd. (Lianyungang, China). Hypertonic citrate adenine solution was sourced from Changzheng Hospital (Shanghai, China). Additional materials comprised a DensiCHEK Plus densitometer (BioMérieux, France), human renal proximal tubular epithelial cell line (HK-2), and CCK-8 assay kit (Solarbio, Beijing, China).

### Bacterial strains

Five clinically representative MDR/XDR bacterial strains were selected for this study: carbapenem-resistant *Klebsiella pneumoniae* (CRKP), carbapenem-resistant *Pseudomonas aeruginosa* (CRPA), carbapenem-resistant *Escherichia coli* (CREC), carbapenem-resistant *Acinetobacter baumannii* (CRAB), and methicillin-resistant *Staphylococcus aureus* (MRSA). MDR was defined as acquired resistance to at least 1 agent in 3 or more antimicrobial categories, XDR was defined as acquired resistance to all but two antimicrobial categories, and pandrug-resistant (PDR) was defined as resistance to all antimicrobial agents currently available for clinical use^32^. All isolates were collected from different inpatients at Shanghai Changhai Hospital. Species identification was conducted using the MALDI Biotyper system (Bruker Daltonics). All methods were performed in accordance with the relevant guidelines and regulations, and experimental protocols were approved by the Ethics Committee of Shanghai Changhai Hospital (Registration No. ChiCTR2200057172). Informed consent was obtained from all subjects.

The use of clinically isolated strains in this study, as opposed to standardized control strains, was intentional to better reflect the current epidemiological landscape and the heterogeneous resistance profiles encountered in real-world clinical practice. This approach enhances the clinical relevance and translational potential of our findings by directly addressing the challenge posed by contemporary MDR/XDR pathogens circulating in healthcare settings.

### Antimicrobial susceptibility testing and detection of resistance genes

The minimum inhibitory concentrations of the tested antibiotics were determined by broth microdilution, with results interpreted according to the Clinical and Laboratory Standards Institute (CLSI) 2024 guidelines^33^. *Escherichia coli* ATCC 25,922 was used as the quality control strain for susceptibility testing. PCR amplification was employed to detect carbapenemase-encoding genes, including *bla*_NDM_, *bla*_KPC_, *bla*_CTX−M_, *bla*_VIM_, and *bla*_OXA−48_. The results of antimicrobial susceptibility testing and the resistance genes detection are summarized in Tables 1 and 2, respectively.

**Table 1:** Antimicrobial susceptibility testing results for each bacterial strain.

Susceptibility	CRPA	CRKP	CREC	CRAB	MRSA
**S**		Tigecycline Polymyxin E	Aztreonam Amikacin Tigecycline Fosfomycin Nitrofurantoin Polymyxin E	Tigecycline TMP-SMX Polymyxin E	Erythromycin Clindamycin Linezolid Vancomycin
**I**			Tobramycin	Minocycline	
**R**	Piperacillin Piperacillin-tazobactam Ceftazidime Cefepime Cefoperazone-sulbactam Imipenem Meropenem Aztreonam Amikacin Tobramycin Gentamicin Levofloxacin Ciprofloxacin	Ampicillin-sulbactam Piperacillin-tazobactam Cefazolin Cefoxitin Ceftriaxone Ceftazidime Cefepime Cefoperazone-sulbactam Ertapenem Meropenem Aztreonam Amikacin Tobramycin Gentamicin Levofloxacin Ciprofloxacin TMP-SMX Fosfomycin	Ampicillin Ampicillin-sulbactam Piperacillin-tazobactam Cefazolin Cefuroxime Cefoxitin Ceftriaxone Ceftazidime Cefepime Cefoperazone-sulbactam Ertapenem Imipenem Meropenem Gentamicin Levofloxacin Ciprofloxacin TMP-SMX	Ampicillin-sulbactam Piperacillin-tazobactam Ceftazidime Cefepime Cefoperazone-sulbactam Imipenem Meropenem Amikacin Tobramycin Gentamicin Doxycycline	Penicillin G Oxacillin Gentamicin Levofloxacin Ciprofloxacin Moxifloxacin Tetracycline TMP-SMX
Cefoxitin screening test					Positive
Inducible clindamycin resistance test					Negative
**CLASS**	PDR	XDR	MDR	XDR	MDR

S, susceptible; I, intermediate; R, resistant; MDR, multidrug-resistant; XDR, extensively drug-resistant; PDR, pandrug-resistant.

**Table 2:** Resistance genes detection results for each strain.

strains	Resistance gene 1	Resistance gene 2
CRPA	VIM	-
CRKP	CTX-M	KPC
CREC	NDM	-
CRAB	OXA-23	-
MRSA	-	-

VIM, Verona integron–encoded metallo-β-lactamase; CTX-M, Cefotaximase-Munich extended-spectrum β-lactamase; KPC, Klebsiella pneumoniae carbapenemase; NDM, New Delhi metallo-β-lactamase; OXA-23, Oxacillinase-23.

### Thermal pretreatment of CMS

A vial containing 150 mg of CMS was reconstituted in 5 ml of PF and serially diluted to achieve a working concentration of 300 mg/L, simulating twice the final concentration. The CMS solution was then subjected to thermal pretreatment in a thermostatic water bath, where it was incubated at either 37–60 °C for 5, 10, 15, or 30 min, resulting in a total of eight distinct pretreatment conditions. After incubation, all samples were cooled to 0–4℃ to eliminate potential temperature-related interference with subsequent assays.

### Inoculum Preparation

After revival and subculture on agar plates, 3–5 well-isolated colonies (> 1 mm in diameter) were picked and suspended in pre-chilled PF. The suspension was adjusted to 0.5 McFarland using a densitometer (approximately 1 ~ 3 × 10^8^ CFU/ml), and then stepwise diluted in pre-cooled PF to obtain a working inoculum of 2 ~ 6 × 10^4^ CFU/ml. All PF used for suspension and dilution was kept at 0 ~ 4°C.

### Simulated hypothermic decontamination and bacterial inoculation

For each bacterial strain, one antibiotic-free blank control group and eight CMS-treated experimental groups were established, each consisting of six replicates. Bacterial suspensions were serially diluted in PF. Aliquots of 50 µl of the diluted suspension were dispensed into a pre-chilled 96-well plate and mixed at a 1:1 ratio with either an equal volume of PF (blank control) or one of the eight preheated CMS samples. The resulting mixture yielded a final bacterial inoculum of approximately 1 ~ 3 × 10⁴ CFU/ml and a CMS concentration of 150 mg/L. The plates were subsequently maintained at 0 ~ 4 °C for 3 h to simulate SCS decontamination.

After decontamination, 10 µl from each well was streaked onto Columbia blood agar plates. The inoculum was spread evenly using a sterile plastic spreader, and the plates were incubated at 37 °C for 20 ~ 24 h. Colony-forming units (CFU) were enumerated, and representative colonies were confirmed by MALDI-TOF mass spectrometry. The decontamination efficacy of each CMS treatment was evaluated by comparing the CFU between each treated group and the blank control group using appropriate statistical methods.

### Cytotoxicity assay on HK-2 cells

HK-2 cells were maintained in complete medium, collected in the exponential growth phase, and seeded into 96-well plates at 5,000 cells/well. CMS was dissolved in serum-free medium (DMEM/F-12) to 150 mg/L, heated at 60 °C for 30 min, and cooled to 0 ~ 4 °C. HK-2 cells were incubated at 0 ~ 4 °C for 3, 6, or 18 h in three groups: background (serum-free medium only), control (cells in serum-free medium), and preheated CMS (cells in serum-free medium containing preheated CMS). Each condition was run in triplicate.

Cell morphology was assessed and recorded using an inverted microscope (40×) and viability was quantified using the CCK-8 assay (450 nm) after background subtraction.

### Data analysis

All statistical analyses were conducted using IBM SPSS Statistics (version 24), and graphs were generated using GraphPad Prism (version 9.5). A two-tailed *P* < 0.05 was considered statistically significant.

A 2 × 4 factorial analysis of variance (factorial ANOVA) was employed to evaluate the main effects of CMS heating temperature (2 levels), heating duration (4 levels), and their interaction. Comparisons between the blank control group and CMS-treated groups were performed based on the distribution and variance homogeneity of CFU data. The specific tests applied were as follows: For data exhibiting normal distribution and homogeneous variances: one-way ANOVA with Tukey’s post hoc test. For data with normal distribution but heterogeneous variances: Welch’s ANOVA with Games–Howell post hoc test. For non-normally distributed data with heterogeneous variances: the Kruskal–Wallis H test with Dunn’s post hoc test.

The average inhibition rate (AIR) was calculated using the formula:

AIR =($\:\overline{\mathrm{C}\mathrm{F}\mathrm{U}}$_blank_-$\:\overline{\mathrm{C}\mathrm{F}\mathrm{U}}$_treated_)/ $\:\overline{\mathrm{C}\mathrm{F}\mathrm{U}}$_blank_. $\:\overline{\mathrm{C}\mathrm{F}\mathrm{U}}$_blank_ and $\:\overline{\mathrm{C}\mathrm{F}\mathrm{U}}$_treated_ represent the mean CFU values of the blank control and each treated group, respectively.

The corrected OD₄₅₀ values (control vs. treatment) at 3, 6, and 18 h were compared by independent samples t-test, as the data met the assumptions of normality and homogeneity of variances.

## Data Availability

All data supporting the findings of this study are available within the paper.
